# Microsatellite instability and mismatch repair gene inactivation in sporadic pancreatic and colon tumours

**DOI:** 10.1038/sj.bjc.6690314

**Published:** 1999-04

**Authors:** C Ghimenti, P Tannergård, S Wahlberg, T Liu, P G Giulianotti, F Mosca, G Fornaciari, G Bevilacqua, A Lindblom, M A Caligo

**Affiliations:** Department of Oncology, Pathology Division, Molecular Genetic Section and Institute of General and Experimental Surgery, University of Pisa, Via Roma 57, 56126 Pisa, Italy; Department of Molecular Medicine, Clinical Genetics, Karolinska Hospital, Stockholm, Sweden

**Keywords:** DNA mismatch repair, *hMLH1* inactivation, *hMSH2* mutations, RER+ tumours

## Abstract

Genomic instability has been proposed as a new mechanism of carcinogenesis involved in hereditary non-polyposis colorectal cancer (HNPCC) and in a large number of sporadic cancers like pancreatic and colon tumours. Mutations in human mismatch repair genes have been found in HNPCC patients, but their involvement in sporadic cancer has not been clarified yet. In this study we screened 21 pancreatic and 23 colorectal sporadic cancers for microsatellite instability by ten and six different microsatellite markers respectively. Microsatellite alterations were observed at one or more loci in 66.6% (14/21) of pancreatic cancers and in 26% (6/23) colon tumours, but all the pancreatic and half of the colon samples showed a low rate of microsatellite instability. All the unstable samples were further analysed for mutations in the *hMLH1* and *hMSH2* genes and for hypermethylation of the *hMLH1* promoter region. Alterations in the *hMLH1* gene were found only in colorectal tumours with a large presence of microsatellite instability. None of the pancreatic tumours showed any alteration in the two genes analysed. Our results demonstrate that microsatellite instability is unlikely to play a role in the tumorigenesis of sporadic pancreatic cancers and confirm the presence of mismatch repair gene alterations only in sporadic colon tumours with a highly unstable phenotype. © 1999 Cancer Research Campaign


*British Journal of Cancer* (1999) **80**(1/2), 11–16
© 1999 Cancer Research Campaign
Article no. bjoc.1998.0314

### Microsatellite instability and mismatch repair gene inactivation in sporadic pancreatic and colon tumours

**C Ghimenti[1,2], P Tannergård[2], S Wahlberg[2], T Liu[2], PG Giulianotti[3], F Mosca[3], G Fornaciari[1], G Bevilacqua[1], A Lindblom[2] and MA Caligo[1]**

[1]Department of Oncology, Pathology Division, Molecular Genetic Section and [3]Institute of General and Experimental Surgery, University of Pisa, Via Roma 57, 56126 Pisa, Italy; [2]Department of Molecular Medicine, Clinical Genetics, Karolinska Hospital, Stockholm, Sweden

**Summary** Genomic instability has been proposed as a new mechanism of carcinogenesis involved in hereditary non-polyposis colorectal cancer (HNPCC) and in a large number of sporadic cancers like pancreatic and colon tumours. Mutations in human mismatch repair genes have been found in HNPCC patients, but their involvement in sporadic cancer has not been clarified yet. In this study we screened 21 pancreatic and 23 colorectal sporadic cancers for microsatellite instability by ten and six different microsatellite markers respectively. Microsatellite alterations were observed at one or more loci in 66.6% (14/21) of pancreatic cancers and in 26% (6/23) colon tumours, but all the pancreatic and half of the colon samples showed a low rate of microsatellite instability. All the unstable samples were further analysed for mutations in the *hMLH1* and *hMSH2* genes and for hypermethylation of the *hMLH1* promoter region. Alterations in the *hMLH1* gene were found only in colorectal tumours with a large presence of microsatellite instability. None of the pancreatic tumours showed any alteration in the two genes analysed. Our results demonstrate that microsatellite instability is unlikely to play a role in the tumorigenesis of sporadic pancreatic cancers and confirm the presence of mismatch repair gene alterations only in sporadic colon tumours with a highly unstable phenotype.

**Keywords:** DNA mismatch repair; *hMLH1* inactivation; *hMSH2* mutations; RER+ tumours

The mismatch repair system is one of the mechanisms that safeguards the fidelity of genetic information since it corrects replicative errors escaping the DNA polymerase proofreading activity (Kolodner, 1996; Modrich and Lahue, 1996). Several human DNA mismatch repair genes have been cloned and involved in hereditary non-polyposis colorectal cancer (HNPCC). These include *hMSH2*, homologue of the bacterial gene *MutS*, which maps on chromosome 2p21–22, and *hMLH1* that maps on chromosome 3p21.3–23 and encodes a homologue of the bacterial MutL and yeast MLH1 proteins (Fishel et al, 1993; Leach et al, 1993; Papadopoulos et al, 1994). The inactive forms of mismatch repair genes might behave as mutator genes causing an accumulation of spontaneous mutations (especially frameshift) at different genetic loci, resulting in a mutator phenotype (Aquilina et al, 1994; Bhattacharyya et al, 1994). This mutator phenotype has been proposed to account for the multiple mutations required in multistage carcinogenesis (Loeb, 1994). An alteration in the length of multiple microsatellite loci, which are sequences particularly prone to slipped-strand mispair because of their repetitive nature, is typical of the mutator phenotype caused by defects of the mismatch repair system (Strand et al, 1993; Dunlop, 1996). Microsatellite instability has been indicated as a replication error positive (RER+) phenotype, pointing out that replicative errors are at the basis of this phenomenon. This instability is the hallmark of colonic and endometrial tumours from HNPCC patients. Several types of sporadic tumours also show an unstable phenotype: e.g. 10–20% of colonic cancer and 60% of pancreatic cancer (Han et al, 1993; Kim et al, 1994). Therefore, it is likely that microsatellite instability is relevant also in the progression of these sporadic tumours.

Interestingly, the incidence of RER appears to vary in different sporadic cancers: colorectal tumours and cancers associated with HNPCC (endometrial and ovary) demonstrate instability at numerous microsatellite loci; on the other hand, other types of sporadic tumours (e.g. lung and breast) show fewer and a less dramatic involvement of microsatellite alterations (Eshleman and Markowitz, 1995; Honchel et al, 1995). Moreover, a comparative study concerning the definition of RER+ phenotypes, has divided sporadic colon tumours into highly unstable (> 20% altered loci) and lowly unstable (< 10% altered loci) ones and observed that only in highly unstable tumours is the normal expression of hMLH1 and hMSH2 lost (Dietmaier et al, 1997).

Current estimates suggest that *hMSH2* and *hMLH1* mutations account for the major fraction of the cancers in HNPCC kindreds (Nystrom-Lahti et al, 1994; Liu et al, 1996). With regard to the sporadic forms of the same tumours, alterations of these genes have been shown in only a few cases of colon, endometrial and ovarian tumours with microsatellite instability (Børresen et al, 1995; Fujita et al, 1995; Katabuchi et al, 1995; Liu et al, 1995; Moslein et al, 1996; Wu et al, 1997). Methylation of the *hMLH1* promoter region has been suggested to be an alternative mechanism of gene inactivation in sporadic colorectal tumours (Kane et al, 1997). However, the proportion and spectrum of sporadic tumours associated with inactivation of mismatch repair genes has not yet been clearly explained.

*Received 25 November 1997*
*Revised 14 October 1998*
*Accepted 20 October 1998*

*Correspondence to:* C Ghimenti

The aim of this study was to analyse the frequency of microsatellite instability in a set of sporadic pancreatic and colonic tumours and to correlate the presence of such phenomenon with inactivation of *hMLH1* and *hMSH2* mismatch repair genes.

### MATERIALS AND METHODS

### Tissues and DNA

Samples (21 pancreatic, 23 colon) of resected tumours and corresponding normal tissue (peripheral blood lymphocytes) were obtained from surgically treated patients at the University of Pisa. DNA was extracted from frozen tissues according to standard protocols (Sambrook et al, 1989).

### Microsatellite analysis

Ten different microsatellite markers were analysed by polymerase chain reaction (PCR) amplification. Two markers were localized on chromosome 2 (D2S313, D2S123); one on chromosome 5 (D5S404); one on chromosome 8 (D8S255); one on chromosome 10 (D10S197); one on chromosome 11 (D11S904) (Weissenbach et al, 1992); and four on chromosome 17q (D17S250, THRA1, D17S579, D17S396) (Futreal et al, 1992; Seifert et al, 1996). All microsatellites were dinucleotide (CA) repeats. The PCR conditions have been described previously (Futreal et al, 1992; Weissenbach et al, 1992; Seifert et al, 1996). A 1 μl aliquot of each PCR reaction was diluted threefold with a buffer consisting of 98% formamide, 20 mM EDTA, 0.05% xilene cyanol, 0.05% bromophenol blue and loaded, after denaturation, on a 6% (38:2) polyacrylamide gel containing 8 M urea. Electrophoresis was conducted at 1400 V for 2–3 h. The gel was dried on 3 MM Whatman paper and exposed to Kodak XAR film at –80°C for 6–12 h. In order to confirm the reproducibility of the experiments, all cases showing microsatellite instability were examined at least twice by an independently performed PCR and electrophoresis.

### Denaturing gradient gel electrophoresis (DGGE)

Each of 19 exons and exon–intron borders of *hMLH1*, and each of 16 exons and exon–intron borders of *hMSH2* (except for exon 5, analysed by constant denaturant gradient gel electrophoresis, CDGE), were screened by DGGE. PCR amplifications of the DNA extracted from tumour samples (and, in some cases, of the normal paired DNA) were performed in a 25-μl volume containing 50 ng template, 5 pmol of each primer, 3.75 nmol dNTP and 0.125 unit of *Taq* polymerase in the buffer provided with different concentrations of magnesium chloride; in some cases, Triton X-100 or glycerol was added to enhance the PCR amplification. Primer sequences and PCR condition have been described previously (Tannergård et al, 1995; Wahlberg et al, 1997).

Gradient gels were made by mixing 100% denaturant (7 M urea and 40% formamide) with 0% denaturant stock solution of 7% acrylamide gels in a gradient maker. Gels were run for 16 h at 85 V, stained with ethidium bromide or silver, and dried on Whatman 3 MM in a vacuum drier. The gradient gel conditions for each exon of both genes were described previously (Tannergård et al, 1995; Wahlberg et al, 1997).

### DNA sequencing

DNA sequencing of PCR products of each analysed exon was performed using cycle sequencing with $^{33}$P-labelled ddNTPs and Thermo Sequenase polymerase (Amersham, Arlington, IL, USA) according to the manufacturer's protocol.

### hMLH1 promoter methylation assay

This protocol was previously described by Kane et al (1997). A total of 250 ng of genomic DNA and 0.004 pg of pRDK447 DNA (a 9.4 kb plasmid containing the yMSH2 gene, a kind gift from Prof. Kolodner) were digested with HpaII or MspI restriction endonucleases in a 20-μl volume reaction. pRDK447 DNA served as internal control for cleavage by HpaII or MspI because it could be cleaved to completion by these enzymes and because the CpG sites in the recognition sequences of these enzymes are not cytosine methylated during propagation in *Escherichia coli*. Reactions containing either no enzyme, 75 units of HpaII, or 150 units of MspI were incubated for 6 h at 37°C.

To analyse cleavage of the *hMLH1* promoter region, 12.5 ng of DNA from each digest were analysed by PCR in 25 μl reaction containing 10 mM Tris-HCL (pH 8.3), 50 mM potassium chloride, 1.5 mM magnesium chloride, 50 mM dNTPs, 0.75 units of AmpliTaq DNA polymerase, and 2.5 pmol of each primer 27494 (5′-CGCTCGTAGTATTCGTGC) and 25266 (5′-TCAGTGC-CTCGTGCTCAC) designed to amplify nucleotides –670 to –67 of *hMLH1*. Importantly for the analysis, HpaII recognition sites were found at nucleotides positions –567, –527, –347 and –341. PCR was performed for one cycle of 95°C for 5 min followed by 33 cycles of 94°C for 30 s, 55°C for 30 s and 72°C for 30 s, followed by one cycle of 72°C for 7 min. The resulting amplification products were then analysed by agarose gel electrophoresis using standard methods.

Cleavage of the control DNA was analysed essentially as described above for the *hMLH1* promoter region, with the following minor modifications. The reactions contained 2.5 pmol of each primer 27859 (5′-TTCTTGGAGGACGACAGC) and 27860 (5′-CAATCACATCTAAATGCG), which amplify a 567-bp piece of *yMSH2* that contains a HpaII site in the middle, in place of the *hMLH1* primers. In addition, the number of amplification cycles was increased to 35.

### RESULTS

### Colon tumours

A total of 23 sporadic colon cancers were analysed for microsatellite alterations using six different markers mapped on six different chromosomes (2, 5, 8, 10, 11, 17). The presence of microsatellite alterations (resulting in expansion or contraction of the number of the repeated units in tumour DNA compared with normal DNA, Figure 1) was observed in six out of 23 tumours (26%). As shown in Table 1, only 50% of these samples demonstrated a high unstable phenotype (more than 40% of altered markers). Tumours with microsatellite alterations were screened by DGGE for *hMLH1* and *hMSH2* gene mutations. One of the high unstable colon tumours (sample NG) showed an altered DGGE pattern in exon 13 of the *hMLH1* gene (Figure 2). No alterations were found in any of the *hMSH2* exons. In order to verify whether the alteration was a germline or a somatic change we also analysed the

 © Cancer Research Campaign 1999

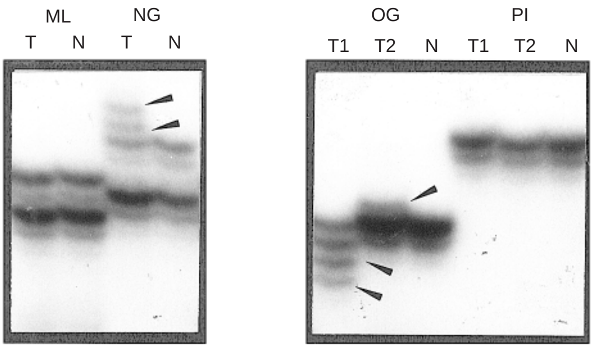

**Figure 1** Examples of microsatellite instability in colon cancer at locus D10S197. Arrows point out the alterations in microsatellite repeats: sample NG shows an expansion in tumour DNA (T) compared with normal DNA (N), sample OG presents a contraction and an expansion in tumours DNA 1 and 2 (T1 and T2) respectively

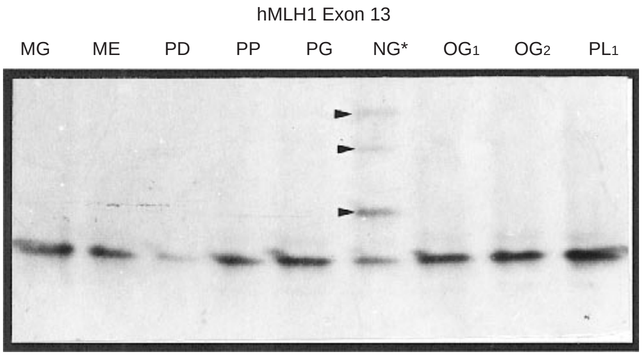

**Figure 2** Denaturing gradient gel electrophoresis of exon 13 of *hMLH1*. The gel has been silver-stained. Colon sample NG shows aberrant bands (pointed out by arrows) in tumour DNA compared with tumour DNA extracted from other patients. The sequence analysis shows that this alteration consists of an 11 bp insertion (TTGTACCCCCC) after the nucleotide number 1489 that caused a premature stop codon TGA after 45 nucleotides normal paired DNA. No alterations in the DGGE pattern could be detected in the normal DNA, confirming the presence of a somatic mutation. The PCR product of the tumour sample showing a DGGE alteration was sequenced in the sense and antisense orientation. The exon 13 alteration in the colon tumour consisted of an 11 bp insertion (TTGTACCCCCC) after the nucleotide 1489 of the cDNA that caused a premature stop codon TGA after 45 nucleotides (Table 2). This mutation has never been described before.

### Pancreatic tumours

A total of 21 pancreatic sporadic cancers were analysed for microsatellite alterations using ten different markers mapped on six different chromosomes (2, 5, 8, 10, 11, 17). The presence of microsatellite alterations was observed in 14 out of 21 tumours (66%). As shown in Table 1, all these samples demonstrated a low unstable phenotype (less than 40% of altered markers). Tumours with microsatellite alterations were screened by DGGE for *hMLH1* and *hMSH2* gene mutations. Only one pancreatic tumour showed an altered DGGE pattern in exon 2 of the *hMLH1*. No alterations were found in any of *hMSH2* exons. In order to verify whether the alteration was a germline or a somatic change we also analysed the normal paired DNA. No alterations in the DGGE pattern could be detected in the normal DNA, confirming the presence of a somatic mutation. The PCR product of the tumour sample showing a DGGE alteration was sequenced in the sense and antisense orientation. The exon 2-altered pattern in the

**Table 1** Selected pancreatic and colon tumours showing microsatellite alterations

| Unstable tumours | Tumour site | Microsatellite probes | | | | | | | | | | Total |
|---|---|---|---|---|---|---|---|---|---|---|---|---|
| | | D2S313 | D2S123 | D5S404 | D8S255 | D10S197 | D11S904 | D17S250 | THRAI | D17S579 | D17S396 | |
| BP | Pancreas | EXP | – | – | – | – | – | – | – | – | – | 1/10 |
| CA | Pancreas | – | – | – | CONTR | – | – | – | – | – | – | 1/10 |
| CI | Pancreas | – | – | CONTR | ND | – | – | EXP | – | EXP | – | 3/9 |
| CR | Pancreas | – | – | ND | – | – | – | – | – | – | EXP | 1/9 |
| FT | Pancreas | EXP | – | – | – | – | – | – | – | ND | – | 1/9 |
| LG | Pancreas | – | – | – | – | – | – | – | – | – | EXP | 1/10 |
| LA | Pancreas | – | – | – | – | – | – | – | – | – | CONTR | 1/10 |
| LF | Pancreas | – | – | – | – | – | – | – | EXP | – | – | 1/10 |
| LD | Pancreas | ND | ND | – | – | – | – | – | EXP | ND | – | 1/7 |
| MG | Pancreas | – | – | – | – | – | – | – | CONTR | – | – | 1/10 |
| ME | Pancreas | – | – | – | – | – | – | – | – | – | CONTR | 1/10 |
| PD | Pancreas | – | – | – | – | – | – | – | – | EXP | – | 1/10 |
| PP | Pancreas | – | EXP | – | – | – | – | – | – | – | CONTR | 2/10 |
| PG | Pancreas | – | ND | – | – | – | – | ND | – | EXP | CONTR | 1/8 |
| NG | Colon | ND | EXP | EXP | EXP | CONTR | EXP | ND | ND | ND | – | 5/6 |
| OG1 | Colon | ND | EXP | CONTR | EXP | CONTR | EXP | ND | ND | ND | – | 5/6 |
| OG2 | Colon | ND | EXP | EXP | CONTR | CONTR | – | ND | ND | ND | – | 4/6 |
| PL1 | Colon | ND | – | – | – | – | CONTR | ND | ND | ND | – | 1/6 |
| PL2 | Colon | ND | – | – | – | – | CONTR | ND | ND | ND | – | 1/6 |
| RA | Colon | ND | – | – | EXP | – | – | ND | ND | ND | – | 1/6 |

CONTR = contraction in the number of microsatellite repeats; EXP = expansion in the number of microsatellite repeats; – = absence of alterations in the number of microsatellite repeats; ND = not done; Total = unstable markers on total number analysed.

**Table 2** hMLH1 alterations found by denaturing gradient gel electrophoresis

| Tumour | Exon affected | Nucleotide change | Predicted protein change |
|---|---|---|---|
| LA | 2 | codon 66 ACC→ACT | None |
| NG | 13 | 11 bp insertion after nucleotide 1489 | Truncation at amino acid 511 |

**Table 3** HNPCC genes alterations and microsatellite instability in pancreatic and colon tumours

| Samples | Tumour site | *hMLH1* gene alterations | *hMSH2* gene mutations | Number of UMM |
|---|---|---|---|---|
| BP | Pancreas | No | No | 1/10 |
| CA | Pancreas | No | No | 1/10 |
| CI | Pancreas | No | No | 3/9 |
| CR | Pancreas | No | No | 1/9 |
| FT | Pancreas | No | No | 1/9 |
| LG | Pancreas | No | No | 1/10 |
| LA | Pancreas | No | No | 1/10 |
| LF | Pancreas | No | No | 1/10 |
| LD | Pancreas | No | No | 1/7 |
| MG | Pancreas | No | No | 1/10 |
| ME | Pancreas | No | No | 1/10 |
| PD | Pancreas | No | No | 1/10 |
| PP | Pancreas | No | No | 2/10 |
| PG | Pancreas | No | No | 1/8 |
| **NG** | **Colon** | **Mutation in the coding region** | **No** | **5/6** |
| **OG1** | **Colon** | **Methylation of the promoter region** | **No** | **5/6** |
| **OG2** | **Colon** | **Methylation of the promoter region** | **No** | **4/6** |
| PL1 | Colon | No | No | 1/6 |
| PL2 | Colon | No | No | 1/6 |
| RA | Colon | No | No | 1/6 |

UMM = unstable microsatellite markers.

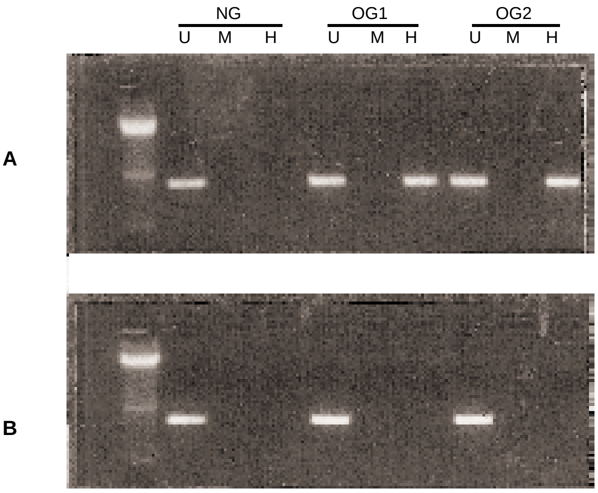

**Figure 3** Analysis of tumour samples for methylation of the *hMLH1* promoter region. (**A**) Amplification of the *hMLH1* promoter region from the indicated tumour DNAs before or after digestion with the indicated restriction endonucleases. (**B**) Amplification of unmethylated internal control DNA before or after digestion with the indicated restriction endonucleases. U, undigested; M, digested with Msp1; H, digested with HpaII. A DNA weight marker has been loaded in the first lane on the left pancreatic tumour resulted in a sequence variant in codon 66 (ACC to ACT) without changes in the coded amino acid (Table 2).

### Polymorphism

Two polymorphisms were found in *hMLH1*. One changed Ile219→Val (CAT to CGT) in exon 8. The other polymorphism was in the intron sequence at position 14 of the splice donor site downstream to exon 13 (G to A). Another two polymorphisms were found in *hMSH2*. The first was a G to T switch at position 12 downstream of the 3′ end of exon 10. The second was a T to G change located 6-bp upstream from the 5′ end of exon 13. All these polymorphisms have been previously described and showed the same patterns of DGGE of the samples studied in this paper (Wijnen et al, 1994; Tannergård et al, 1995; Wahlberg et al, 1997). A comparison was done processing our samples with positive controls with known polymorphisms; all the samples always showed the same DGGE patterns.

### Methylation of the hMLH1 promoter region

The methylation status of the hMLH1 promoter in pancreatic and colon samples displaying alterations of microsatellite markers was examined using a PCR assay. The result of this analysis showed that only the *hMLH1* promoter region from the two high unstable colon tumours with no alteration in the *hMLH1* coding region (samples OG1 and OG2) was methylated: in both cases the promoter region was resistant to digestion by HpaII and sensitive to digestion by MspI (Figure 3 and Table 3). In all the experiments, the unmethylated internal control DNA was sensitive to digestion by both enzymes.

### DISCUSSION

Fidelity of DNA replication is crucial in avoiding the accumulation of mutations in critical genes and an excessive global mutational load. *hMSH2*, *hMLH1*, *hPMS1* and *hPMS2* are members of two highly conserved families of post-replication mismatch repair genes, *MutS* and *MutL* (Modrich and Lahue, 1996). Functional disruption of *MutS*, *MutL*, or of their homologues, in bacteria and yeast increases the rate of spontaneous mutations resulting in a mutator phenotype (Aquilina et al, 1994; Bhattacharyya et al, 1994; Eshleman et al, 1995). In particular, mutations of these genes cause a random effect of instability in anonymous sequences made of di-tri-tetra nucleotide repeats. Microsatellite unstable cancers were first detected in HNPCC in 1993 (Aaltonen et al, 1993). Since then a large number of studies have demonstrated the presence of genomic instability in a wide cohort of sporadic and familial tumours (reviewed by Eshleman and Markowitz, 1995; Honchel et al, 1995). In this study, 21 pancreatic cancers and 23 colon tumours were analysed to evaluate the incidence of genomic instability.

Microsatellite instability was found in six out of 23 (26%) colon tumours, but half of them showed alterations at only one locus out of six loci analysed. Our findings are in good agreement with previous studies (Ionov et al, 1993; Kim et al, 1994). The six RER+ colon tumours were screened for mutations in the *hMSH2* and *hMLH1* genes. No mutations in the *hMSH2* gene, and only one somatic mutation in the *hMLH1* gene, were found. Our results are in good agreement with previous studies that demonstrated that

 © Cancer Research Campaign 1999

mismatch repair gene mutations are very rare in the RER+ sporadic forms of colon cancers: the *hMSH2* gene was found to be altered in only nine out of 62 colon tumours (Børresen et al, 1995; Liu et al, 1995; Wu et al, 1997); the *hMLH1* gene was found to be mutated in only eight out of 50 colon cancers (Liu et al, 1995; Moslein et al, 1996, Wu et al, 1997).

Fourteen out of 21 (66.6%) pancreatic tumours were RER+, but 86% (12/14) were altered at a single locus out of the ten analysed; those samples should probably be classified as stable tumours. *hMLH1* and *hMSH2* genes were not found to be altered in any of those unstable tumours. Pancreatic cancer is a rare disease and only two studies analysing a limited number of cases have been published, both reporting an incidence of this phenomenon as low as the one we found. In the first, two out of six pancreatic tumours were unstable at only one locus out of the three tested (Han et al, 1993). In the second, one out of five pancreatic tumours was unstable at one out of the 12 loci tested (Brentnall et al, 1995). Moreover, this low level of instability is frequently found in other gastrointestinal cancers such as gastric and oesophageal tumours (Keller et al, 1995; Muzeau et al, 1997). Our results support the hypothesis that the genetic mechanism of carcinogenesis in sporadic pancreatic cancer is not likely to be linked to microsatellite instability.

The low rate of mutations detected in our cohort cannot be explained by relative low sensitivity of the method because the DGGE technique is a highly sensitive method and can detect > 90% of small base sequence alterations, such as splice site, missense, non-sense and frameshift mutations (Fodde and Losekoot, 1994; Myers et al, 1987). Nevertheless, large deletions and mutations in the region that the primers hybridize may not be detected by this technique.

Recently, *hMLH1* promoter region methylation has been proposed as an alternative mechanism by which mismatch repair genes are silenced (Kane et al, 1997). In our study, we analysed the methylation status of the *hMLH1* promoter region in all the tumour samples showing alteration of microsatellite markers. Two colon samples showing a high unstable phenotype and no mutation in the coding region of the *hMLH1* gene displayed resistance to HpaII digestion, a hallmark for cytosine methylation of CpG islands.

It is therefore interesting to observe that all the colon samples showing more than 40% of unstable loci show defects in the mismatch repair gene (mutation in the coding sequence or inactivation by promoter region methylation). These data are in agreement with a comparative study carried out by Dietmaier and co-workers (1997): colon tumour samples showing less than 20% of altered markers could not be considered truly unstable and display a normal expression of *hMSH2* and *hMLH1*.

It has been hypothesized that inactivation of both alleles of the mismatch repair gene is required to generate an unstable phenotype (Leach et al, 1993; Parsons et al, 1993; Liu et al, 1996). We were unable to demonstrate an altered methylation of the promoter region of the *hMLH1* gene in the colon sample showing only the somatic mutation of the gene. However, we could not exclude that the second allele is inactivated by other mechanisms, such as large deletion or that the found mutation could be dominant-negative like the truncating mutation at codon 134 of the *hPMS2* mismatch repair gene (Nicolaides et al, 1998).

### ACKNOWLEDGEMENTS

This work was supported by UICC (Union Internationale Contre le Cancer), by AIRC (Italian Association for Cancer Research), by CNR (Centro Nazionale delle Ricerche), target project ACRO 9500309.PF39, and by the Swedish Cancer Society.

### REFERENCES

Aaltonen LA, Peltomäki P, Leach FS, Sistönen P, Pylkkänen L, Mecklin J, Järvinen H, Powell SM, Jen J, Hamilton SR, Petersen GM, Kinzler KW, Vogelstein B and De la Chapelle A (1993) Clues to the pathogenesis of familial colorectal cancer. *Science* **260**: 812–816

Aquilina G, Hess P, Branch P, MacGeoch C, Casciano I, Karran P and Bignami M (1994) A mismatch recognition defect in colon carcinoma confers DNA microsatellite instability and a mutator phenotype. *Proc Natl Acad Sci USA* **91**: 8905–8909

Bhattacharyya NP, Skandalis A, Ganesh A, Groden J and Meuth M (1994) Mutator phenotypes in human colorectal carcinoma cell lines. *Proc Natl Acad Sci USA* **91**: 6319–6123

Børresenn A-L, Lothe RA, Meling GI, Lystad P, Morrison P, Lipford J, Kane MF, Rognum TO and Kolodner RD (1995) Somatic mutations in the hMSH2 gene in microsatellite unstable colorectal carcinomas. *Hum Mol Genet* **4**: 2065–2072

Brentnall, TA, Chen R, Lee JG, Kimmey MB, Bronner MP, Haggitt RC, Kowdley KV, Hecker LM and Byrd DR (1995) Microsatellite instability and K-ras mutations associated with pancreatic adenocarcinoma and pancreatitis. *Cancer Res* **55**: 4264–4267

Dietmaier W, Wallinger S, Bocker T, Kullmann F, Fishel R and Rüschoff J (1997) Diagnostic microsatellite instability: definition and correlation with mismatch repair protein expression. *Cancer Res* **57**: 4749–4756

Dunlop MG (1996) Mutator genes and mosaicism in colorectal cancer. *Curr Opin Genet Dev* **6**: 75–80

Eshleman JR and Markowitz SD (1995) Microsatellite instability in inherited and sporadic neoplasms. *Curr Opin Oncol* **7**: 83–89

Eshleman JR, Lang EZ, Bowerfind GK, Parsons R, Vogelstein B, Willson JKV, Veigl ML, Sedwick WD and Markowitz SD (1995) Increased mutation rate at the hprt locus accompanies microsatellite instability in colon cancer. *Oncogene* **10**: 33–37

Fishel RA, Lescoe MK, Rao MRS, Copeland N, Jenkins N, Garber J, Kane M and Kolodner R (1993) The human mutator gene homolog MSH2 and its association with hereditary non-polyposis colon cancer. *Cell* **75**: 1027–1038

Fodde R and Losekoot M (1994) Mutation detection by denaturing gradient gel electrophoresis (DGGE). *Hum Mutat* **3**: 83–94

Fujita M, Enomoto T, Yoshino K, Nomura T, Buzard GS, Inoue M and Okudaira Y (1995) Microsatellite instability and alterations in the hMSH2 gene in human ovarian cancer. *Int J Cancer* **64**: 361–366

Futreal PA, Soderkvist P, Marks JR, Inglehart JD, Cochran C, Barrett JC and Wiseman RW (1992) Detection of frequent allelic loss on proximal chromosome 17q in sporadic breast carcinoma using microsatellite length polymorphisms. *Cancer Res* **52**: 2624–2627

Han HJ, Yanagisawa A, Kato Y, Park JG and Nakamura Y (1993) Genetic instability in pancreatic cancer and poorly differentiated type of gastric cancer. *Cancer Res* **53**: 5087–5089

Honchel R, Halling KC and Thibodeau SN (1995) Genomic instability in neoplasia. *Semin Cell Biol* **6**: 45–52

Ionov Y, Peinado MA, Malkhosyan S, Shibata D and Perucho M (1993) Ubiquitous somatic mutations in simple repeated sequences reveal a new mechanism for colonic carcinogenesis. *Nature* **363**: 558–561

Kane MF, Loda M, Gaida GM, Lipman J, Mishra R, Goldman H, Jessup JM and Kolodner R (1997) Methylation of the hMLH1 promoter correlates with lack of expression of hMLH1 in sporadic colon tumors and mismatch repair-defective human tumor cell lines. *Cancer Res* **57**: 808–811

Katabuchi H, Van Rees B, Lambers AR, Ronnett BM, Blazes MS, Leach FS, Cho KR and Hedrick L (1995) Mutations in DNA mismatch repair genes are not responsible for microsatellite instability in most sporadic endometrial carcinomas. *Cancer Res* **55**: 5556–5560

Keller G, Roter M, Vogelsang H, Bischoff P, Becker K-F, Mueller J, Brauch H, Siewert JR and Höfler H (1995) Microsatellite instability in adenocarcinomas of the upper gastrointestinal tract. *Am J Pathol* **147**: 593–600

Kim H, Jen J, Vogelstein B and Hamilton SR (1994) Clinical and pathological characteristics of sporadic colorectal carcinomas with DNA replication errors in microsatellite sequences. *Am J Pathol* **145**: 148–156

Kolodner R (1996) Biochemistry and genetics of eukaryotic mismatch repair. *Genes Dev* **10**: 1433–1442

Leach FS, Nicolaides NC, Papadopoulos N, Liu B, Jen J, Parsons RE, Peltomäki P, Sistönen P, Aaltonen LA, Nyström-Lahti M, Guan XY, Zang J, Meltzer PS, Yu

© Cancer Research Campaign 1999

JW, Kao FT, Chen DJ, Cerosaletti KM, Fournier REK, Todd S, Lewis T, Leach RG, Naylor SL, Weissenbach J, Mecklin J-P, Järvinen H, Petersen GM, Hamilton SR, Green J, Jass J, Watson P, Lynch HT, Trent JM, De la Chapelle A, Kinzler KW and Vogelstein B (1993) Mutations of a mutS homolog in hereditary non-polyposis colorectal cancer. *Cell* **75**: 1215–1225

Liu B, Nicolaides NC, Markowitz S, Willson JKV, Parsons RE, Jen J, Papadopoulos N, Peltomaki P, De la Chapelle A, Hamilton SR, Kinzler KW and Vogelstein B (1995) Mismatch repair gene defects in sporadic colorectal cancers with microsatellite instability. *Nature Genet* **9**: 48–55

Liu B, Parsons R, Papadopoulos N, Nicolaides NC, Lynch HT, Watson P, Jass JR, Dunlop M, Wyllie A, Peltomäki P, De la Chapelle A, Hamilton SR, Vogelstein B and Kinzler KW (1996) Analysis of the mismatch repair genes in the hereditary non-polyposis colorectal cancer patients. *Nature Med* **2**: 169–174

Loeb LA (1994) Microsatellite instability: marker of a mutator phenotype in cancer. *Cancer Res* **54**: 5059–5063

Modrich P and Lahue R (1996) Mismatch repair in replication fidelity, genetic recombination, and cancer biology. *Ann Rev Biochem* **65**: 101–133

Moslein G, Tester DJ, Lindor NM, Honchel R, Cunningham JM, French AJ, Halling KC, Schwab M, Goretzki P and Thibodeau SN (1996) Microsatellite instability and mutation analysis of hMSH2 and hMLH1 in patients with sporadic, familial and herediatary colorectal cancer. *Hum Mol Genet* **5**: 1245–1252

Muzeau F, Flejou J-F, Belghiti J, Thomas G and Hamelin R (1997) Infrequent microsatellite instability in oesophageal cancers. *Br J Cancer* **75**: 1336–1339

Myers RM, Maniatis T and Lerman LS (1987) Detection and localization of single base changes by denaturating gradient gel electrophoresis. *Nucleic Acids Res* **17**: 501–528

Nicolaides NC, Littman SJ, Modrich P, Kinzler KW and Vogelstein B (1998) A naturally occurring hPMS2 mutation can confer a dominant negative mutator phenotype. *Mol Cell Biol* **18**: 1635–1641

Nyström-Lahti M, Parsons R, Sistönen P, Pylkkänen L, Aaltonen LA, Leach FS, Hamilton SR, Watson P, Bronson E, Fusaro R, Cavalieri J, Lynch J, Lanspa S, Smyrk T, Lynch P, Drouhard T, Kinzler KW, Vogelstein B, Lynch HT, De la Chapelle A and Peltomäki P (1994) Mismatch repair genes on chromosomes 2p and 3p account for major share of hereditary non-polyposis colorectal cancer families evaluable by linkage. *Am J Hum Genet* **55**: 659–665

Papadopoulos N, Nicolaides NC, Wei Y, Ruben SN, Carter KC, Rosen CA, Haseltine WA, Fleischmann RD, Fraser CN, Adams, MD, Venter JC, Hamilton SR, Petersen GM, Watson P, Lynch HT, Peltomaki P, De la Chapelle A, Kinzler KW and Vogelstein B (1994) Mutation of mutL homolog in hereditary colon cancer. *Science* **263**: 1625–1629

Parsons R, Li G-M, Longley M, Fang WH, Papadopoulos N, Jen J, de la Chapelle A, Kinzler KW, Vogelstein B and Modrich P (1993) Hypermutability and mismatch repair deficency in RER+ tumor cells. *Cell* **75**: 1227–1236

Sambrook J, Fritsch EF and Maniatis T (1989) *Molecular Cloning: a Laboratory Manual*, 2nd Edn. Cold Spring Harbor Laboratory Press: Cold Spring Harbor

Seifert M, Seib T, Engel M, Dooley S and Welter C (1996) Characterization of the human NM23.H2 promoter region and localization of the microsatellite D17S396. *Biochem Biophys Res Commun* **215**: 910–914

Strand M, Prolla TA, Liskay RM and Petes TD (1993) Destabilization of tracts of simple repetitive DNA in yeast by mutations affecting DNA mismatch repair. *Nature* **365**: 274–276

Tannergård P, Lipford JR, Kolodner R, Frödin JE, Nordenskjöld M and Lindblom A (1995) Mutation screening in the hMLH1 gene in Swedish hereditary nonpolyposis colon cancer families. *Cancer Res* **55**: 6092–6096

Wahlberg S, Nyström-Lahti M, Kane MF, Kolodner RD, Peltomäki P and Lindblom A (1997) Few mutations in the hMSH2 gene in Swedish HNPCC families found by denaturing gradient gel electrophoresis. *Int J Cancer* **74**: 134–137

Weissenbach J, Gyapay G, Dib C, Vignal A, Morissette J, Milasseau P, Vaysseix G and Lathrop MA (1992) A second-generation linkage map of the human genome. *Nature* **359**: 794–801

Wijnen J, Fodde R and Kahn PM (1994) DGGE polymorphism in intron 10 of MSH2, the HNPCC gene. *Hum Mol Genet* **3**: 2263–2268

Wu Y, Nyström-Lahti M, Osinga J, Looman MWG, Peltomäki P, Alatonen LA, de la Chapelle A, Hofstra RMW and Buys CHCM (1997) MSH2 and MLH1 mutations in sporadic replication error-positive colorectal carcinoma as assessed by two-dimensional DNA electrophoresis. *Gene Chrom Cancer* **18**: 269–278

 *© Cancer Research Campaign 1999*
